# Safety and Efficacy of Double-J Metallic Mesh Ureteral Stents in Malignant Ureteral Obstruction

**DOI:** 10.7759/cureus.84072

**Published:** 2025-05-14

**Authors:** Sho Nonoyama, Jun Furumido, Ryo Kato, Nariaki Ozaki, Yasukuni Matsugase

**Affiliations:** 1 Department of Urology, Asahikawa Kosei General Hospital, Hokkaido, JPN

**Keywords:** double-j metallic ureteral stent, double j ureteral stent, malignant ureteral obstruction, metallic stent, stainless steel mesh ureteral stent

## Abstract

Introduction: The mortality rate of malignant ureteral obstruction (MUO) is high, and ureteral stents are widely used for its treatment. Polymeric ureteral stents are commonly utilized, but stent occlusion due to tumor obstruction poses a problem. Conversely, metallic ureteral stents, while pressure resistant, can cause significant pain during insertion and replacement, often necessitating anesthesia. Since 2017, double-J metallic mesh ureteral stents have been on the market, but reports on their efficacy and safety are still limited. We collected data on using this stent at our hospital and conducted an analysis and evaluation to report our findings.

Methodology: We retrospectively studied 23 cases in which stainless mesh ureteral stents were inserted at our hospital from April 2023 to December 2024.

Results: A total of 29 ureters were stented in 23 cases (14 females and 9 males). The mean age was 69 years (range = 46-81 years), with primary diseases including gynecological cancer in 11 cases, gastrointestinal cancer in seven cases, urological cancer in four cases, and one case of retroperitoneal fibrosis. Four cases required stent replacement due to stent failure during the observation period. The mean follow-up period was 400 days, during which eight cases died due to the primary disease.

Conclusions: We examined the use of double-J metallic mesh ureteral stents at our hospital. The stents showed high utility for MUO. Furthermore, our findings suggest that they may reduce the burden on both physicians and patients, as they potentially eliminate the need for frequent replacements compared to polymeric ureteral stents, which typically require replacement every three months.

## Introduction

Ureteral obstructions secondary to malignant tumors have a high mortality rate, and ureteral stents are widely used for their treatment. While polymeric ureteral stents are frequently employed for this condition, they present several challenges, including a high stent failure rate and the need for frequent replacements, which can result in secondary trauma [1]. Therefore, it is essential to select methods with higher patency rates and longer durations than those offered by conventional stents.

Double-J metallic ureteral stents are among the latest treatment options for managing malignant ureteral obstruction (MUO). They offer high resistance to external compression and provide a longer duration of use compared to polymeric stents [2]. Currently, double-J metallic ureteral stents are made of a double-J metallic coil either integrated as the stent material itself (Resonance®, Cook Medical, Bloomington, IN) or embedded within the stent wall (Tumor DD Ureter Stent, Rüsch, Teleflex Medical, Wayne, PA) [3]. The Resonance® stent is resistant to external compression forces, offering more strength that is at least three to four times greater than conventional polymeric stents [4]. Despite previous studies indicating high patency rates, the Resonance® stent lacks a central lumen due to its structural design and requires a specialized technique for proper placement [5].

Another type of metallic ureteral stent, known as the Tumor DD Ureter Stent, is a double-J metallic mesh stent that has been available on the market since 2017. This is a hydrophilic-coated stent made of a polymeric tube reinforced with stainless steel mesh. It not only offers the same level of strength and durability as the Resonance® stent but also features a central lumen, allowing it to be implanted in the same manner as standard polymeric stents [2].

However, since this is a relatively new device, research on its use remains limited. We believe that further investigation is crucial for determining the justification of using double-J metallic mesh ureteral stents for managing MUO.

The present study aimed to analyze the efficacy and safety of the use of the double-J metallic mesh ureteral stent for MUO based on data we collected at Asahikawa Kosei General Hospital.

## Materials and methods

A retrospective review of medical records at a single institution was performed. A total of 23 patients who were treated with the double-J metallic mesh ureteral stent (Tumor DD Ureter Stent, Rüsch, Teleflex Medical) at Asahikawa Kosei General Hospital from April 2023 to December 2024 were potentially eligible for the study. Bilateral ureters in the same patient were counted as separate ureteral units. The collected medical data included age, gender, follow-up duration in months, laterality, primary disease, laboratory data, existence of previous polymeric ureteral stent, time to replacement, and complications.

The operational method for inserting the double-J metallic mesh ureteral stent is similar to that of ordinary polymeric ureteral stents. The stent lengths used for each case were 22, 24, and 26 cm, while the diameter was 7 Fr in all cases. The placement procedure was performed using the same method as conventional stents. Specifically, a cystoscope was used to insert a guidewire through the ureteral orifice, followed by retrograde pyeloureterography to assess the stricture site and ureteral length. After confirmation, an appropriately sized double-J metallic mesh ureteral stent was placed in a retrograde manner. Stent replacement was also performed following the same procedure under local anesthesia.

The outcomes assessed were stent failure, stent patency rate, and overall survival. Stent failure was defined as unscheduled stent exchange or nephrostomy tube placement for signs or symptoms of ureteral obstruction. Stent patency was defined as either complete or partial resolution of hydronephrosis and preexisting renal dysfunction, or maintenance of renal function in cases that had previously been treated with polymeric stents. Overall survival was defined as the time from the double-J metallic mesh ureteral stent placement to final follow-up or death and calculated using the Kaplan-Meier method.

The mental workload associated with the insertion of the stent was assessed using a questionnaire completed by five doctors. The questionnaire included six indices based on the National Aeronautics and Space Administration-Task Load Index (NASA-TLX). NASA-TLX is a widely used, validated measure of self-reported workload [6-7]. The instrument assesses perceived mental, physical, and temporal demands as well as effort, performance, and frustration associated with a job task. A higher NASA-TLX score indicates greater perceived workload and stress experienced by the operator.

All statistical analyses were performed with EZR (Saitama Medical Center, Jichi Medical University, Saitama, Japan), which is a graphical user interface for R (The R Foundation for Statistical Computing, Vienna, Austria). More precisely, it is a modified version of R Commander designed to add statistical functions frequently used in biostatistics [8].

The Wilcoxon signed-rank test was used to compare serum creatinine level (sCre) and estimated glomerular filtration rate (eGFR) before and after stenting, and *P* < 0.05 was considered statistically significant.

## Results

A total of 29 stents were inserted in 23 patients (14 females, 9 males) since 6 patients had bilateral stents. The 6 patients with bilateral stents had previously been managed with polymeric stents before being replaced with double-J metallic mesh ureteral stents. The clinical characteristics of the 23 patients are summarized in Table 1. The median age of the patients was 69 years (range = 46-81). Endometrial cancer was the most frequent type of malignancy (5 patients, 21.7 %), followed by cervical cancer (4 patients, 17.4 %).

**Table 1:** Baseline characteristics.

Baseline characteristics		*n* (%) or (median, range)
Age (years)		69 (46 - 81)
Sex	Female	14 (60.9)
	Male	9 (39.1)
Follow-up duration in months (median, range)		13.4 (4.0-19.7)
Laterality	Unilateral	17 (73.9)
	Bilateral	6 (26.1)
Primary disease	Endometrial cancer	5 (21.7)
	Cervical cancer	4 (17.4)
	Ovarian cancer	2 (8.7)
	Prostate cancer	2 (8.7)
	Rectal cancer	2 (8.7)
	Sigmoid colon cancer	2 (8.7)
	Stomach cancer	2 (8.7)
	Bladder cancer	1 (4.3)
	Pancreatic cancer	1 (4.3)
	Renal pelvis and ureteral cancer	1 (4.3)
	Retroperitoneal fibrosis	1 (4.3)

Table 2 shows the outcome during the observational period for the patients who had double-J metallic mesh ureteral stent placement. Stent failure occurred in four ureters (13.8%): as shown in Table 3, all four ureters developed hydronephrosis, and three ureters developed fever. Among the four cases, three were managed with stent exchange: two using the double-J metallic mesh ureteral stent and one using the Resonance® stent, while one case underwent stent removal due to calcification.

**Table 2:** Types and numbers of outcomes in patients treated with double-J metallic mesh ureteral stents.

		*n* (%)
Death	Dead	8 (34.8)
	Alive	15 (65.2)
Replacement due to stent failure	Yes	4 (13.8)
	No	25 (86.2)
Unscheduled admission	Yes	5 (21.7)
	No	18 (78.3)

**Table 3:** Four cases with stent occlusion.

Primary disease	Sex	Age (years)	Time to replacement (months)	Symptoms
Ovarian cancer	Female	80	4.4	Fever, hydronephrosis
Endometrial cancer	Female	73	19.2	Fever, hydronephrosis
Cervical cancer	Female	52	18.6	Hematuria, hydronephrosis
Gastric cancer	Male	46	9.0	Fever, hydronephrosis

The frequency and type of complications are summarized in Table 4. Urosepsis and cystitis occurred in two cases each (8.7%), while acute renal failure, hematuria, iliopsoas abscess, and urinary incontinence were each observed in one case (4.3%). Patients with urosepsis, acute renal failure, hematuria, and iliopsoas abscess after placement of the double-J metallic mesh ureteral stent required unscheduled admission.

**Table 4:** Types and numbers of complications in patients treated with double-J metallic mesh ureteral stents.

	*n* (%)
Urosepsis	2 (8.7)
Cystitis	2 (8.7)
Acute renal failure	1 (4.3)
Hematuria	1 (4.3)
Iliopsoas abscess	1 (4.3)
Urinary incontinence	1 (4.3)
No complications	15 (65.2)

**Figure 1:**
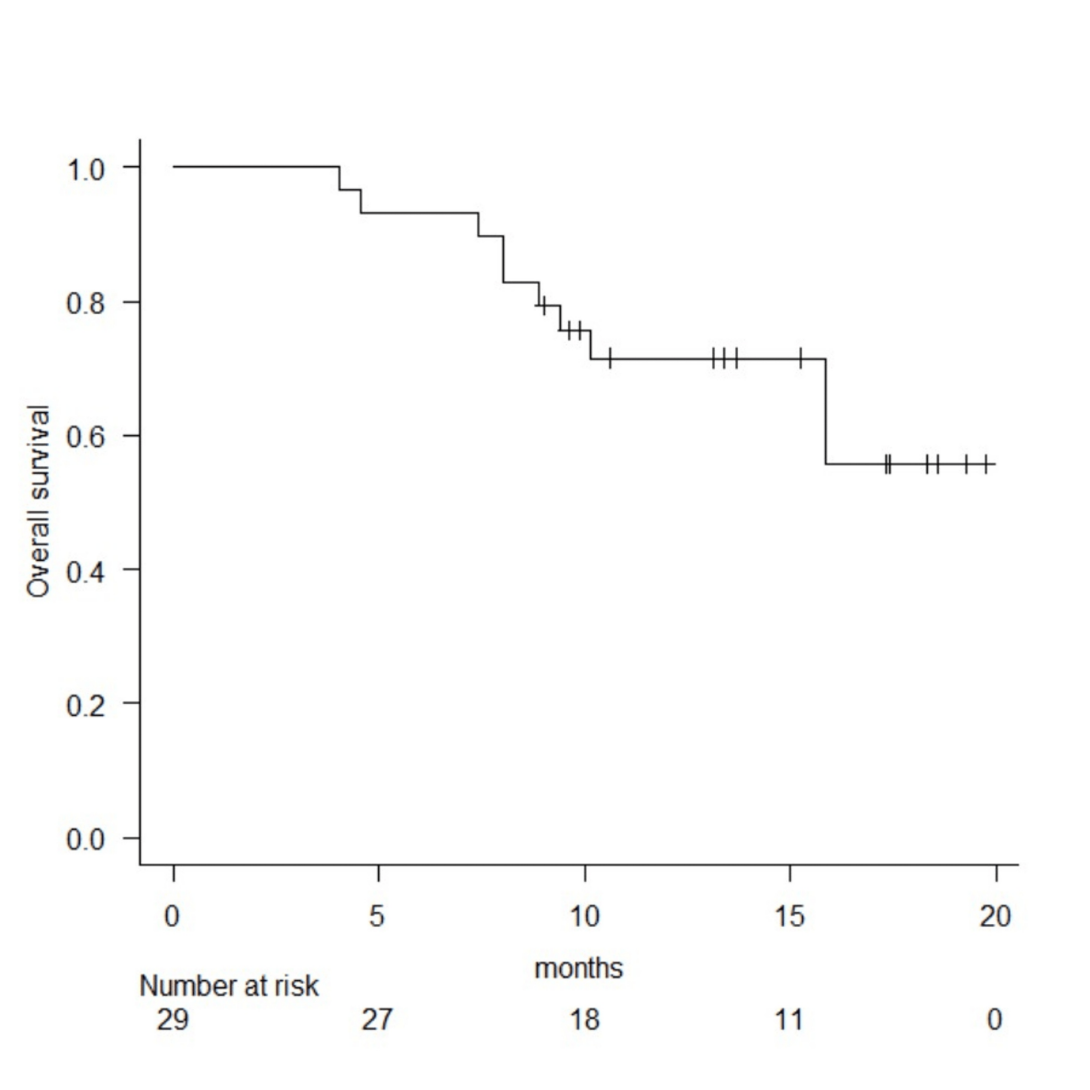
Estimated overall survival after double-J metallic mesh ureteral stent placement.

The median observation period was 13.4 months (range = 4.0-19.7 months), and the median overall survival was not reached at the time of analysis (Figure 1).

**Figure 2:**
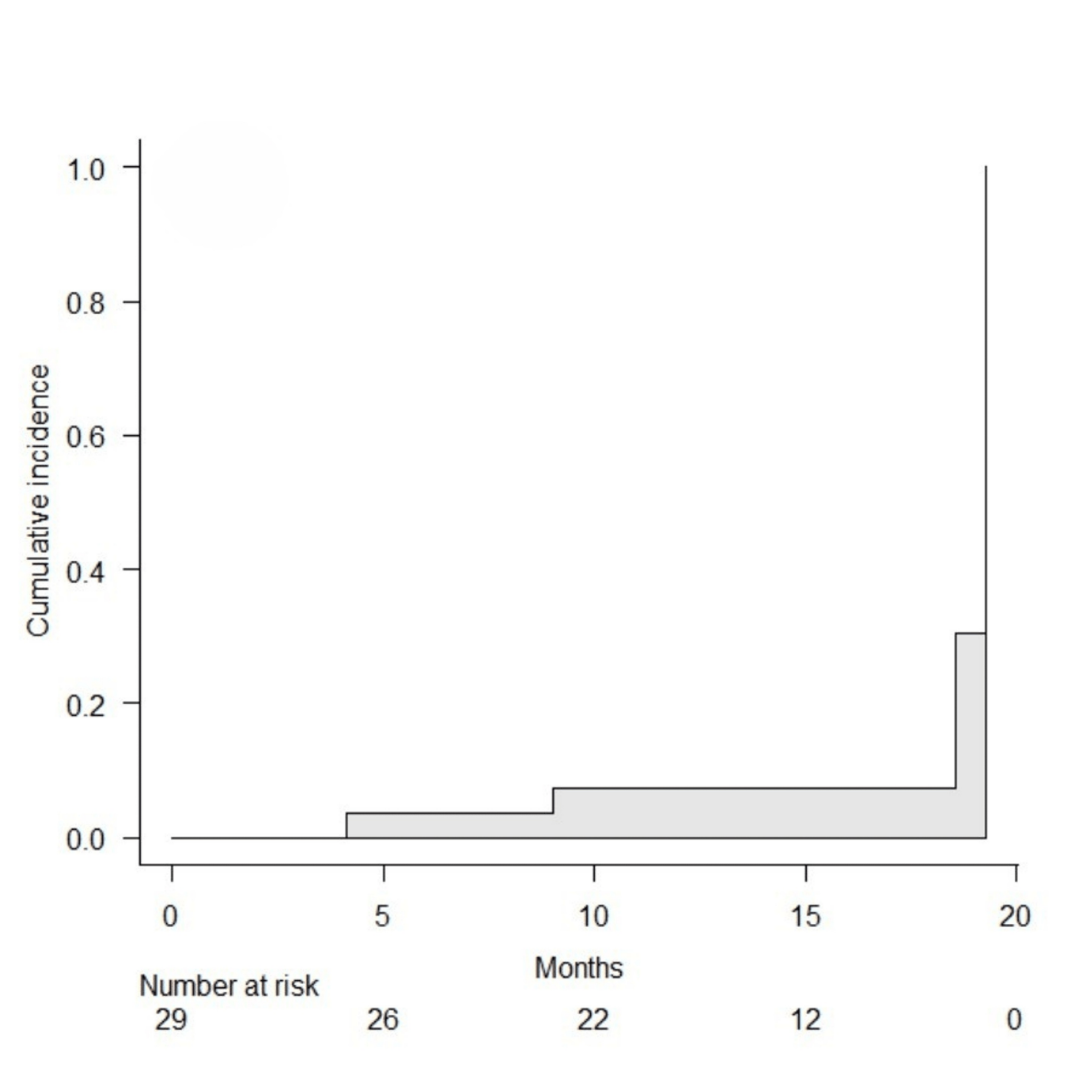
Cumulative incidence curve of stent failure after double-J metallic mesh ureteral stent placement.

The cumulative incidence of stent failure at 3, 6, and 12 months after the first stent placement was 4.5% (95% confidence interval (CI) = 0.7%-28.1%), 9.3% (95% CI = 2.4%-32.4%), and 13.8% (95% CI = 5.3%-25.8%), respectively (Figure 2).

**Figure 3:**
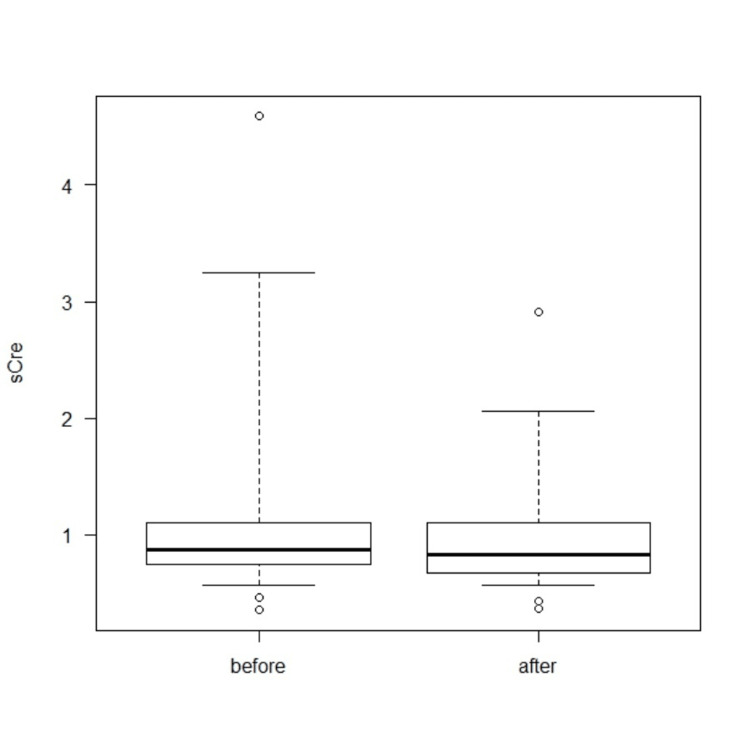
Serum creatinine (sCre) transition before and after double-J metallic mesh ureteral stent insertion.

**Figure 4:**
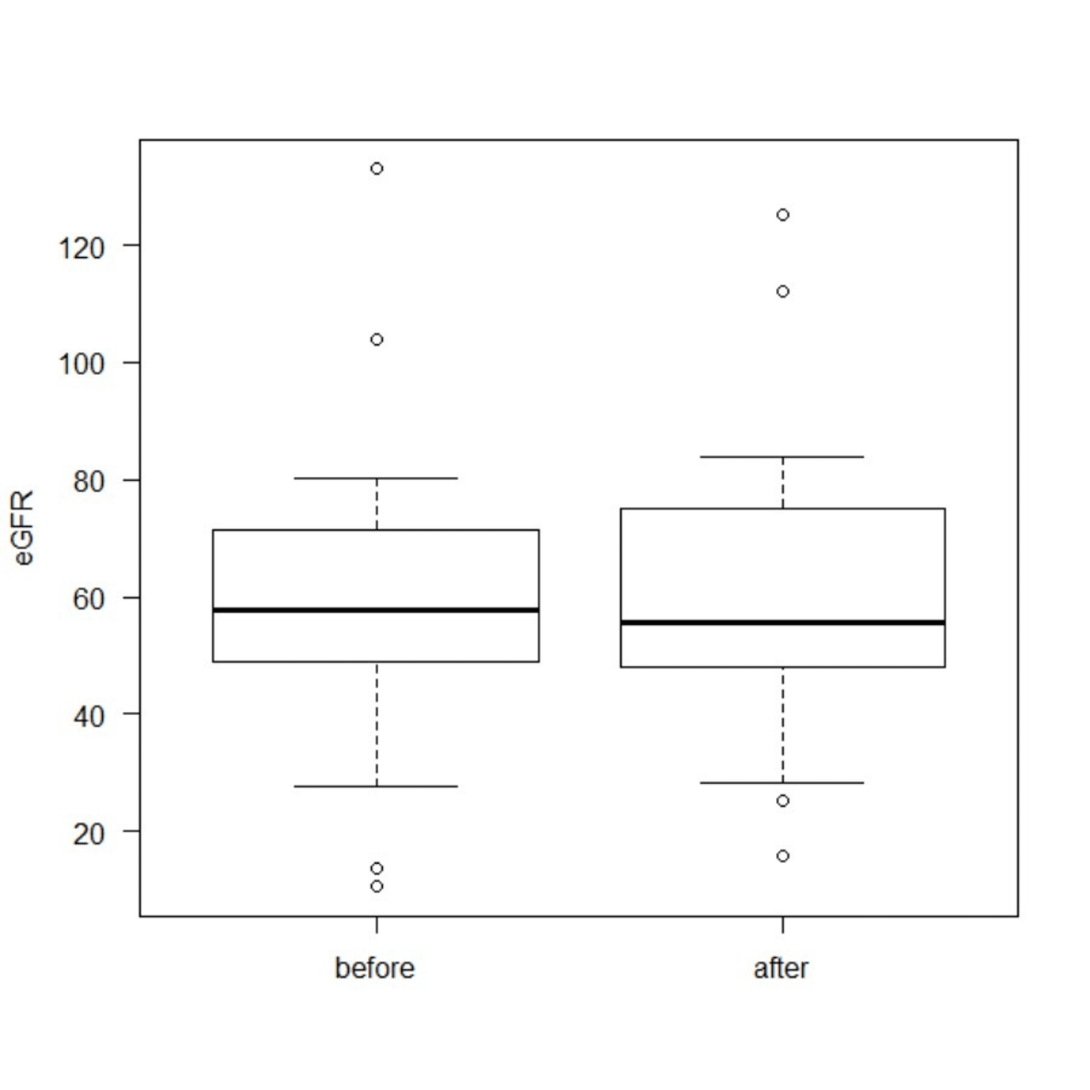
eGFR transition before and after double-J metallic mesh ureteral stent insertion. eGFR, estimated glomerular filtration rate

The median serum creatinine (sCre) level before stenting was 0.87 mg/dL (range: 0.4-4.5 mg/dL), compared to 0.83 mg/dL (range: 0.4-2.9 mg/dL) following stenting (*P* = 0.673; Figure 3). The median eGFR before stenting was 57.8 mL/min/1.73 m² (range: 10.5-133), and 55.7 mL/min/1.73 m² (range: 15.9-125.5) after stenting (*P* = 0.933; Figure 4).

**Figure 5:**
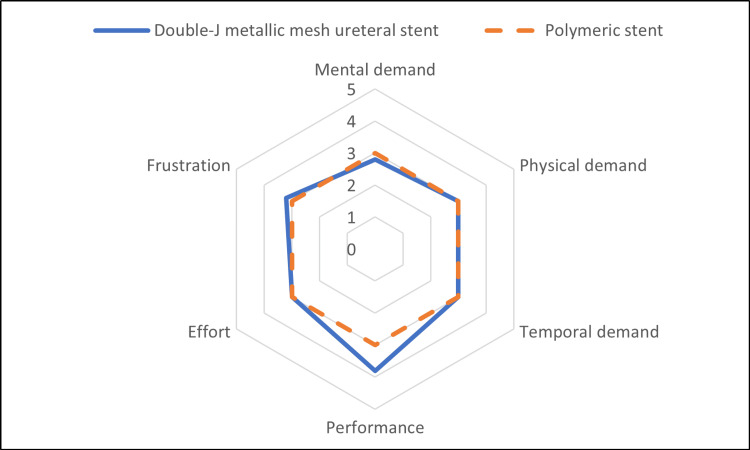
Mental workload for double-J metallic mesh ureteral stent insertion. Comparison of the NASA-TLX scores with those of polymeric stents. NASA-TLX, National Aeronautics and Space Administration-Task Load Index

We sent out questionnaires of NASA-TLX to five urologists. All five urologists have had several experiences of inserting both polymeric and the double-J metallic mesh ureteral stent. The average workload score in six subscales was analyzed (Figure 5). The double-J metallic mesh ureteral stent achieved a higher performance score; however, no significant difference was observed when compared to polymeric ureteral stents.

## Discussion

As treatment options for malignant ureteral obstruction, ureteral stent placement and nephrostomy are available, with ureteral stent placement being the more commonly chosen option. Nephrostomy is considered in cases where ureteral stent placement is unsuccessful or when stent dysfunction occurs, as it significantly reduces the patient’s quality of life. Traditional polymer stents show a successful placement rate of approximately 85% and a stent failure rate of about 20% [9]. Generally speaking, polymeric stents are recommended to be exchanged every 6-12 weeks [10-12]. However, the short interval necessitates frequent exchanges, which could increase the risk of secondary trauma for patients and impose a heavy burden on doctors.

In this study, we investigated the safety and efficacy of the double-J metallic mesh ureteral stents (Tumor DD Ureter Stent, Rüsch, Teleflex Medical) for MUO. The double-J metallic mesh ureteral stent is a long-term, indwelling ureteral stent that was approved for insurance coverage in 2017. It is a polymer-based ureteral stent reinforced with a double-J metallic mesh, offering high resistance to ureteral compression caused by tumors and safe for MRI up to 3 Tesla [13].

Currently, an older type of double-J metallic stent is available: the double-J metallic ureteral stent made of a metallic coil, integrated as the stent material itself (Resonance®, Cook Medical).

It is more commonly used, and multiple studies have shown its effectiveness [1,14-18]. Previous research reported that the cumulative incidence of stent failure at 12 months after placement was 18.8% (95% CI = 6.1%-29.7%) [14,19].

This study found that the double-J metallic mesh ureteral stent demonstrated a favorable cumulative stent failure rate of 13.8% (95% CI = 5.3%-25.8%) at 12 months after placement. These results were comparable to those of the Resonance® stent. Moreover, double-J metallic mesh ureteral stents may offer greater utility, as their lumen allows a guidewire to pass through, enabling implantation and replacement in the same manner as a conventional polymeric ureteral stent [2].

Although the optimal indwelling period of double-J metallic mesh ureteral stent is unclear, it is marketed by the manufacturer as being suitable for indwelling for up to 12 months. Additionally, a 12-month period seems reasonable given the low stent failure rate and the fact that the Resonance® stent has an anticipated indwelling period of 12 months [20]. In the present study, 7 out of 23 patients died within 12 months of their first double-J metallic mesh ureteral stent placement.

A previous study showed that the median survival time of MUO patients was 210 days [14]. Our present analysis revealed the median survival time of 344 days. Although there are some differences between the studies, the survival time of MUO patients is generally expected to be quite short in clinical settings. Given that the median survival time for MUO is less than 12 months, stent replacement may not be necessary when using double-J metallic mesh ureteral stents.

Additionally, double-J metallic mesh ureteral stents can be replaced under local anesthesia regardless of the patient's sex, making it a less invasive option for patients compared to the Resonance® stent, which sometimes requires spinal anesthesia to exchange [14, 17, 19]. In our study population, two cases required replacement with double-J metallic mesh ureteral stents, and the exchanges were successfully performed under local anesthesia with no complications.

Furthermore, the double-J metallic mesh ureteral stent is easier to replace compared to the Resonance® stent and the polymeric stent for the following reasons. Prior research has indicated that replacing the Resonance® stent can be challenging due to its structural differences [5]. In contrast, the insertion and replacement process of double-J metallic mesh ureteral stents is similar to that of traditional polymeric stents, and our NASA-TLX assessment results demonstrated that exchanging them did not increase the mental workload on doctors. One study reported that 35% of polymeric stents could not be exchanged after stent failure, theoretically due to complete obstruction of the stent lumen [14]. In our study, the percentage of unexchangeable double-J metallic mesh ureteral stents was lower, with only one out of four cases (25%), which may be attributed to its resistance to compression forces.

This study has several limitations. Its retrospective design may introduce selection bias and limit the ability to establish causal relationships, and the limited sample size may affect the generalizability of the findings.

Moreover, severe hydronephrosis and the absence of treatment after stent placement have been reported as prognostic factors for stent failure in polymeric stents [21-22]. Further research is needed to evaluate these factors regarding double-J metallic mesh ureteral stents.

## Conclusions

In summary, we conducted a retrospective analysis using data of patients with MUO to investigate the efficacy and safety on the use of a double-J metallic mesh ureteral stent. A double-J metallic mesh ureteral stent is considered highly useful for both doctors and patients due to its high patency rate and suitability for a long-term placement. Our findings suggest it may offer a durable and practical alternative for managing MUO in clinical settings.
